# Identification of a new R3 MYB type repressor and functional characterization of the members of the MBW transcriptional complex involved in anthocyanin biosynthesis in eggplant (*S*. *melongena* L.)

**DOI:** 10.1371/journal.pone.0232986

**Published:** 2020-05-14

**Authors:** Moglia Andrea, Elia Florio Francesco, Iacopino Sergio, Guerrieri Alessandra, Anna Milani Maria, Comino Cinzia, Barchi Lorenzo, Marengo Arianna, Cagliero Cecilia, Rubiolo Patrizia, Toppino Laura, Leonardo Rotino Giuseppe, Lanteri Sergio, Bassolino Laura

**Affiliations:** 1 Department of Agricultural, Forest and Food Sciences, Plant Genetics and Breeding, University of Torino, Grugliasco (TO), Italy; 2 CREA, Research Centre for Genomics and Bioinformatics, Montanaso Lombardo (LO), Italy; 3 PlantLab, Scuola Superiore Sant'Anna, Institute of Life Sciences, Ghezzano (PI), Italy; 4 Department of Biology, University of Pisa, Pisa (PI), Italy; 5 Plant Hormone Biology Group, Swammerdam Institute for Life Sciences (SILS), University of Amsterdam, Amsterdam, The Netherlands; 6 Department of Drug Science and Technology, University of Torino, Torino (TO), Italy; 7 CREA, Research Centre for Cereal and Industrial Crops, Bologna, Italy; University of Naples Federico II, ITALY

## Abstract

Here we focus on the highly conserved MYB-bHLH-WD repeat (MBW) transcriptional complex model in eggplant, which is pivotal in the transcriptional regulation of the anthocyanin biosynthetic pathway. Through a genome-wide approach performed on the recently released Eggplant Genome (cv. 67/3) previously identified, and reconfirmed by us, members belonging to the MBW complex (*SmelANT1*, *SmelAN2*, *SmelJAF13*, *SmelAN1*) were functionally characterized. Furthermore, a regulatory R3 MYB type repressor (*SmelMYBL1*), never reported before, was identified and characterized as well.

Through a qPCR approach, we revealed specific transcriptional patterns of candidate genes in different plant tissue/organs at two stages of fruit development. Two strategies were adopted for investigating the interactions of bHLH partners (*Smel*AN1, *Smel*JAF13) with MYB counterparts (*Smel*ANT1, *Smel*AN2 and *Smel*MYBL1): Yeast Two Hybrid (Y2H) and Bimolecular Fluorescent Complementation (BiFC) in *A*. *thaliana* mesophylls protoplast. Agro-infiltration experiments highlighted that *N*. *benthamiana* leaves transiently expressing *SmelANT1* and *SmelAN2* showed an anthocyanin-pigmented phenotype, while their co-expression with *SmelMYBL1* prevented anthocyanin accumulation. Our results suggest that *SmelMYBL1* may inhibits the MBW complex *via* the competition with MYB activators for bHLH binding site, although this hypothesis requires further elucidation.

## Introduction

Anthocyanins are the major plant flavonoid compounds, which confer appealing colours to flowers and fruits and contribute to stress tolerance [1,2]. In plant vegetative tissues, anthocyanins play key roles in protection against UV radiation, low/high temperatures, drought and pathogen attacks, while in reproductive organs they exert also an eco-physiological role by attracting pollinators and seed dispersers. Anthocyanins also possess widely documented antioxidant, antidiabetic, antihyperlipidemic, anti-inflammatory, anticarcinogenic properties and a preventive activity against cardiovascular diseases in humans [3].

Eggplant purple fruits are a rich source of anthocyanins, being delphinidin-3-*p*-coumaroylrutinoside-5-glucoside and the delphinidin-3-rutinoside the most abundant [4]. The anthocyanin pathway represents one branch of flavonoid metabolism and it is a very conserved network in many plant species, with most of the genes encoding for enzymes and regulatory transcription factors (TFs) identified in several plant species [5]. The anthocyanin pathway is one of the most finely tuned and it is under the control of Early (EBGs) and Late (LBGs) Biosynthetic Genes in dicotyledonous species [6,7]. *Chalcone synthase* (*CHS)*, *Chalcone-flavonone isomerase* (*CHI)*, *Flavanone 3-hydroxylase* (*F3H)* and *Flavonol synthase* (*FLS)* are common EBGs involved in the biosynthesis of all downstream flavonoids, whose enzymatic steps are controlled by co-activator independent and functionally redundant ‘R2R3-type MYB’ regulatory genes (*MYB11*, *MYB12*, *MYB111*) [8].

*Flavonoid 3' hydroxylase* (*F3՛H)*, *Flavonoid-3'*,*5'-hydroxylase* (*F3՛5՛H)*, *Dihydroflavonol 4-reductase* (*DFR)*, Anthocyanidin synthase (*ANS*) are LBGs required for anthocyanin synthesis and modification, and their correlation with anthocyanin content has been highlighted in many *Solanaceous* species including eggplant [6,9–11]. RNAseq analyses showed that most of the eggplant LBGs were up-regulated in flower and young fruit skin tissues at the early stage fruit development of ripening, with a marked decrease at the physiological stage of ripening [12].

It has been reported that the activation of LBGs is mediated by the MYB-bHLH-WD40 (MBW) transcription complexes. MYB proteins, together with bHLH and WD40, can act as positive or negative transcriptional regulators binding to the promoters of structural genes.

The MYB family is one of the largest in flowering plants, with 125 members in *A*. *thaliana* [13] sharing common features at amino terminus: the DNA binding domain consists of one to three conserved HLH motifs, referred as R1, R2, R3. The MYB activators mainly belong to the R2R3-MYB family of transcription factors, while repressors consist of both R2R3-MYB and R3-MYB. It has been proposed the involvement of diverse MBW complexes depending on the occurrence of activator or repressor MYBs which directly, and competitively, bind the bHLH *via* the amino terminus domain, acting in a tissue-specific mode to modulate anthocyanin synthesis [14]. Indeed, the MBW complex is counterbalanced by the amount of MYB repressor(s) which inactivate the complex by recruiting the bHLH partner [5]. The WD40 proteins (*e*.*g*. PhAN11) modulate the activity of MYB/bHLH regulators at post-transcriptional level and there are no evidences of a direct interaction with these TFs [15].

In the *Solanaceae*, the genes encoding R2R3-MYB transcription factors are orthologs of the petunia *PhAN2* [16], while those encoding IIIf group of bHLHs are orthologs of the two petunia groups: the *AN1* and the *JAF13* [17,18]; the physical interaction of PhAN1 and PhAN2 proteins is required to activate the transcription of LBGs (*e*.*g*. *DFR*) [17]. In eggplant, the orthologs of tomato *ANT1* and *AN2* [19], belonging to R2R3-MYBs, have been identified and found to be preferentially expressed in the early fruit maturation stage as well as in flowers [12]. The transient expression of *SmelANT1* in tobacco [12, 20] and the stable expression into a non-anthocyanin accumulating eggplant [9,20, 21] both lead to anthocyanin synthesis, suggesting its role in controlling fruits and flower pigmentation. The orthologs of tomato *AN1* and *JAF13* belonging to bHLH family have been identified in eggplant as well [12].

The role of MYB repressors has only recently being recognized thanks to the studies in petunia, grapevine, poplar and tomato [22–25]. The R2R3-MYBs can act as both positive and negative transcriptional regulators. Indeed, the petunia R2R3-type *Ph*MYB27 acts as repressor of anthocyanin biosynthetic pathway, since its overexpression leads to a reduced anthocyanin content [5,26]. Recently, a R3-MYB encoding gene with three DNA-binding domain repeats, namely *ATROVIOLACEA* (*ATV*), has been characterized in tomato [25]; however, genes encoding MYB repressors have not been yet identified in eggplant, pepper, and potato.

Recently a high quality, annotated and anchored eggplant genome sequence (www.eggplantgenome.org; [12]) has been made available, leading to the identification of genes of MBW complex (*Smel*ANT1, *Smel*AN2, *Smel*JAF13 and *Smel*AN1), in the present paper we confirmed their sequence homology also by functional domain identification and phylogenetic analyses. Furthermore, we identified, for the first time in eggplant, a regulatory R3 MYB repressor according to its high similarity with MYBL1, recently described in the genus Iochroma (*Solanaceae*) by Gates and co-workers [27]. The expression dynamics of candidate genes were assessed at two stages of fruit development and in flowers. To the best of our knowledge, this is the first report on the establishment of eggplant interaction of MYB proteins with bHLH partners, which we assessed both in a yeast two‐hybrid system and *via* Bimolecular Fluorescent Complementation (BiFC) in *A*. *thaliana* mesophyll protoplasts. At last, we estimated the effect of over-expression of the candidate genes in agro-infiltrated *N*. *benthamiana* plants and established their effect on anthocyanin content.

## Materials and methods

### Plant material and growth conditions

Plants of *S*. *melongena* line “67/3” with violet-black round fruits, were grown in pots (30 cm diameter) in glasshouse at CREA (Montanaso Lombardo, Lodi—Italy), under standard conditions, from March to September 2017. For each organ (open flowers, fruits), samples were obtained by pooling tissues collected from at least 3 plants. At least one flower per plant was collected at anthesis (S1A Fig). Skin and flesh of the fruits were collected at the unripe (stage A, S1B Fig) and commercial ripening stage (stage B, S1C Fig) according to Mennella et al. [4]. All fresh tissues were immediately frozen in liquid nitrogen and stored at -80°C.

### Phylogenetic analysis and identification of regulatory protein

The deduced amino acid sequences of bHLHs and MYBs were obtained by screening the genome of the eggplant breeding line 67/3 [20] using the pBLAST function tool of Eggplant Genome Browser (http://www.eggplantgenome.org/), with the amino acid sequences of petunia and tomato regulatory anthocyanin proteins as query (S1 and S2 Files).

Sequence alignment of bHLH and MYBs from eggplant as well as from known anthocyanin related bHLH and MYB in other species (from NCBI and Sol Genomic Network (https://solgenomics.net) (S1 and S2 Files) were generated via multiple sequence alignment using the ClustalW algorithm in the MEGA X package [28].

The evolutionary history of both families of TFs was visualized by the Neighbour-Joining method via MEGA X. All ambiguous positions were removed for each sequence pair (pairwise deletion option) resulting in 1301 (for bHLH tree) and 2561 (for MYB tree) positions in the final dataset. The statistical significance of individual nodes was assessed by bootstrap analysis with 1,000 replicates, and the evolutionary distances were calculated using the p-distance method with default parameters. A second round of alignment and phylogenetic analysis was performed, as described above, with a limited number of MYBs belonging to known flavonoids repressor proteins.

### MYBL1 structure analysis

The nucleotide sequence data of *SmelMYBL1* (*SMEL*_010g336390.1) is available at GeneBank Database with accession number MN855525. The Intron/Exon organization for *SmelMYBL1* gene was determined by aligning the cDNA sequences to their corresponding genomic DNA sequences used as the input for graphical display at the Gene Structure Display Server of Peking University, China (http://gsds.cbi.pku.edu.cn/). Moreover, the protein sequence of *Smel*MYBL1 was aligned to other similar MYBL1 proteins using ClustalW in MEGA software.

### Real-time PCR analysis

Total RNA from eggplant cv. 67/3 tissues was extracted using the TRIzol RNA Isolation Reagents (Thermo Fisher Scientific) combined with the Spectrum Plant Total RNA kit (Sigma Aldrich). The single strand cDNA was synthesized from 1 μg of RNA using a High Capacity RNA-to-cDNA kit (Applied Biosystems, Foster City, USA). Amplifications were performed with primers designed by Primer 3 software (frodo.wi.mit.edu/cgi-bin/primer3/primer3 www.cgi) for eggplant *AN2*, *ANT1*, *AN11*, *JAF13*, *AN1*, *DFR*, *MYBL1*. All the gene-specific primer sequences are listed in S1 Table. A standard amplification curve was generated for each gene using 2-fold serial dilution of pooled cDNA. PCR efficiency was optimized to be in the range 80–100% with R^2^-values of 0.996. The PCR reactions were carried out using the Rotor-Gene RG-6000 thermal cycler (Corbett Research) according to the following PCR parameters 95°C for 5 min, followed by incubation for 15s at 95°C and denaturation for 15 s at 95°C, annealing for 60 s at 59°C for 40 cycles, followed by elongation at 72°C for 20 s. Specificity of amplifications was assessed by melt curves analyzed for the presence of a single peak. The analyses were performed on three biological replicates and in technical triplicates. Expression of *Smel*GAPDH [29] was used as reference gene. Relative expression levels of each individual gene were calculated using GeNorm (https://genorm.cmgg.be/) [30].

### Cloning of the MYBs and bHLHs encoding genes

*Smel*ANT1, *Smel*AN2, *Smel*MYBL1, *Smel*JAF13, *Smel*AN1 were first amplified from cDNA using primers with attB1 and attB2 sites, cloned by Gateway Recombinant Technology in pDONOR 207 vector through a BP recombination and subsequently transferred by LR recombination into the destination vector, as described in the following paragraphs.

### Yeast two-hybrid

ProQuest™ Two-Hybrid System (Life Technologies), kindly provided by Dr. Montanini from University of Parma, was used. Each entry vector (pDONOR207 with the CDS of the gene of interest as detailed above) was recombined with the activation domain (AD) vector pDEST22 (for ANT1, AN2 and MYBL1) and/or the binding-domain (BD) vector pDEST32 (for JAF13 and AN1). *S*. *cerevisiae* strain Mav203 was transformed with 1μg each of the different combinations of bait, prey and control (non-recombined) vectors using the lithium acetate/polyethylene glycol method. Transformed colonies containing bait and prey plasmids were selected on synthetic dropout medium lacking Tryptophan and Leucine (−W/−L). To test the interaction between bait and prey, an equal number of cells was spotted on medium lacking Tryptophan, Leucine and Uracil (−W/−L/−U). Negative controls using empty vectors were also performed.

Two round-shaped 50 mm Whatman 541 filter papers, saturated with 3.5 ml of 2% X-gal solution, were placed on a 10 cm petri dish. The transformed yeasts from the surface of the YPAD plates were obtained by a 50 mm Whatman 541 filter paper and then completely immersed in liquid nitrogen for 15 seconds and then set them on the top of the soaked Whatman filters. Plates were sealed with a parafilm and incubated at 37°C for overnight. The results were recorded after 1 hours by photography.

### Bimolecular Fluorescent Complementation (BiFC) analysis

*A*. *thaliana* Columbia-0 ecotype mesophyll protoplasts were isolated and transformed as previously described [31]. AN1 full CDS entry vector was recombined with N9842 vector [32] to generate AN1-nYFP fusion protein. AN2, ANT1 and MYBL1 full CDS were instead recombined with the N9843 vector [32] to generate AN2-cYFP, ANT11-cYFP and MYBL1-cYFP fusion proteins, respectively. Both N9842 and N9843 vectors were kindly provided by Dr. Beatrice Giuntoli (Department of Biology, University of Pisa). As negative control, plasmids containing the expression cassette for nYFP-GUS or cYFP-GUS fusion proteins [33] were used in combination with the previously described vectors. Plasmid DNA was isolated using a DNA Maxi-prep kit. Protoplasts were transformed using 2.5 μg of each plasmid and stained with 2 μg of DAPI (Sigma-Aldrich). Confocal investigation was performed with the Zeiss AiryScan confocal microscope. YFP fluorescent was exited with a 488 nm laser and collected at 490–540 nm. Chlorophyll fluorescent was exited with 640 nm laser and collected at 650–750 nm. DAPI was exited at 405 nm and collected at 410–470 nm. Images were analysed with the ZEN 2010software (Zeiss).

### Transient heterologous expression in *N*. *benthamiana*

For transient expression, the pEAQ-HT vector kindly provided by Prof. Lomonossoff [34], was used. Each entry vector (pDONOR207 with the CDS of the gene of interest as detailed above) was then recombined with the pEAQ-HT destination vector. The pEAQ-HT destination vectors (containing genes of interest) as well as the empty vector pEAQ-HT, used as a negative control, were inserted in *Agrobacterium tumefaciens* strain C5801 by the freeze-thaw method. Transformed bacteria were grown overnight at 28°C in 5 mL of L medium (10g L^-1^ bactotryptone, 5 g L^-1^ Yeast extract, 5 g L^-1^ NaCl, 1 g L^-1^ D-glucose) containing kanamycin (50 mg L^-1^). The overnight cultures (2 mL) were then transferred into 20 mL of induction medium (L broth containing 10 mM MES and 20 μM acetosyringone) with kanamycin (50 mg L^-1^),and grown as above. The cells were collected by centrifugation for 10 min at 4,000 g and resuspended in 50 mL of infiltration medium (10 mM MgCl_2_, 10 mM MES, 200 μM acetosyringone) to an OD_600_ of 1.0 and kept at room temperature for 3 h before being infiltrated into the abaxial air spaces of 2–4-week-old *N*. *benthamiana* plants. After 4 days, the infiltrated leaf material was collected and used for quantitative HPLC/PDA analysis as described below.

### Identification and quantification of anthocyanins

Transiently transformed *N*. *benthamiana* grinded tissues (1 g) were extracted with 8 mL EtOH/HCl (85/15), pH = 1. After centrifugation (10,000 g for 10 min), supernatants were obtained.

The identification and quantification of delphinidin 3-*O*-rutinoside was carried out by HPLC on a Shimadzu XR system equipped with a photodiode detector SPD-M20A (Shimadzu, Dusseldorf Germany). HPLC-grade acetonitrile (HPLC plus ≥99.9%) and formic acid (> 98% purity) were purchased from Sigma Aldrich (Bellefonte, USA). De-ionized water (18.2MΩ cm) was obtained from a Milli-Q purification system (Millipore, Bedford, MA, USA). Delphinidin 3-*O*-rutinoside was obtained from Extrasynthese (Genay Cedex, France).

Each acid extract prepared was filtered with a 13 mm diameter, 0.22 μm pore diameter hydrophilic PTFE syringe filter and then analyzed on an Ascentis Express C18 column (15 cm ×2.1 mm, 2.7 μm, Supelco, Bellefonte, USA) using water/formic acid (99:1, v/v) and acetonitrile/formic acid (99:1, v/v) as mobile phases A and B, respectively. The flow rate was 0.4 mL min^-1^ and the column temperature was maintained at 30°C. The gradient program was as follows: 5% B for 15 min, 5–20% B in 5 min, 20–100% B in 6 min, 100% B for 2 min. Total pre-running and post-running time was 36 min. UV spectra were acquired over the 220–700 nm wavelength range. The quantification of delphinidin 3-*O*-rutinoside (Retention Time: 19.155 min) in the extracts was performed through the external calibration method at 520 nm. The calibration curve (S2 Fig) of the authentic commercial standard was prepared with six different concentrations, in the 50–1 μg mL^-1^ range (curve equation: y = 14200x-13960, R^2^ = 0.9996). All the data were statistically analyzed using SPSS statistical software.

## Results and discussion

### Identification of eggplant anthocyanin-related regulatory genes

Based on a genome-wide phylogenetic approach, we identified eggplant putative MYB and bHLH transcription factors as candidates for anthocyanin regulation. The bHLH encoding genes were spotted by screening the genome of *S*. *melongena* cv. 67/3 [12] with 13 known plant proteins related to anthocyanin synthesis belonging to the subgroup IIIf [35] (S1 File), whose members are known to be involved in flavonoid regulations.

**Fig 1:**
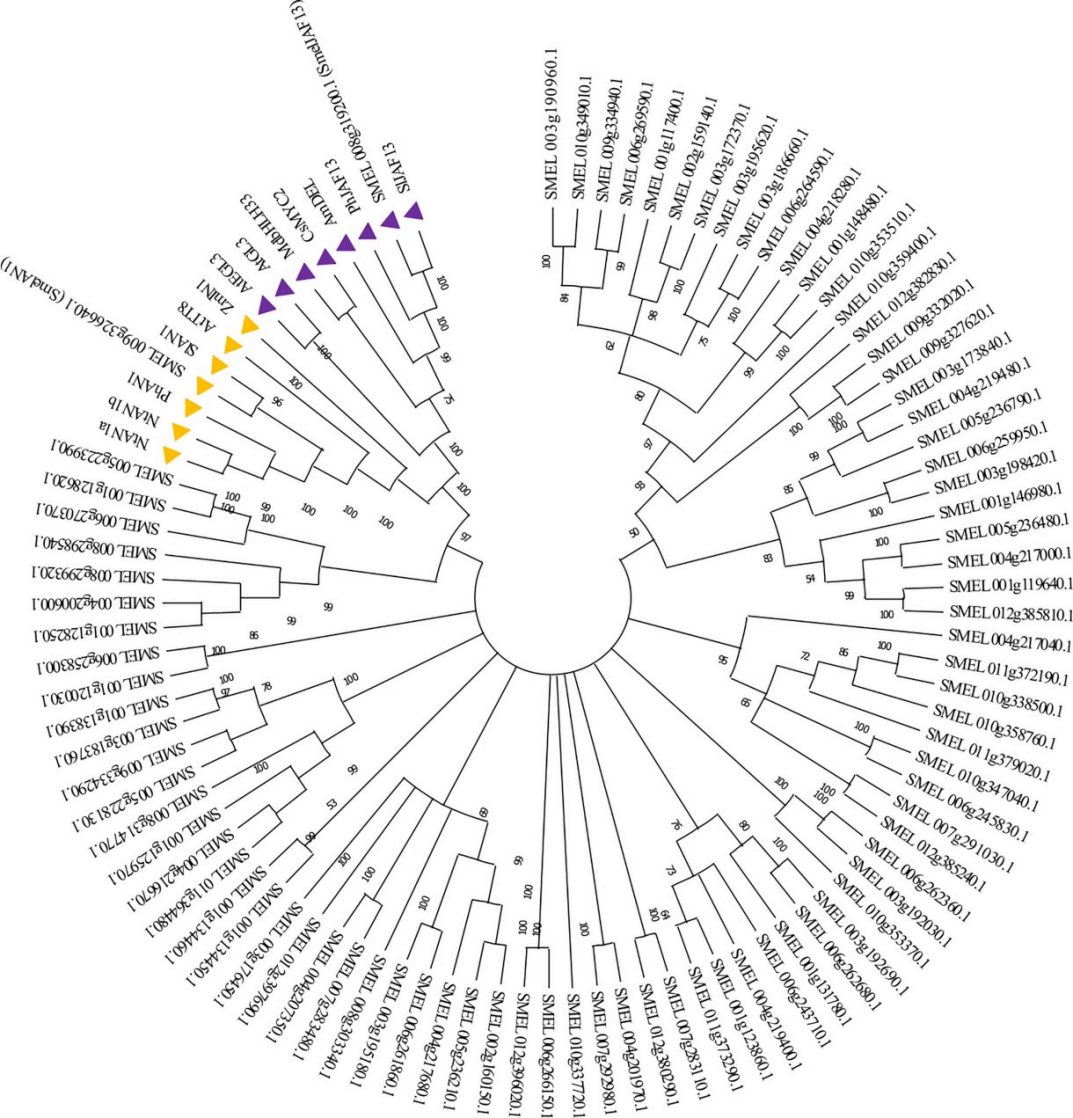
Phylogenetic tree of bHLH transcription factors in *S*. *melongena* genome. The optimal NJ tree with the sum of branch length = 31.39844401 is shown. The tree is drawn to scale, with branch lengths in the same units as those of the evolutionary distances used to infer the phylogenetic tree. The percentage of replicate trees in which the associated taxa clustered together in the bootstrap test (1,000 replicates) are shown next to the branches. Branches corresponding to partitions reproduced in less than 50% bootstrap replicates are collapsed. The evolutionary distances were computed using the p-distance method and are in the units of the number of amino acid differences per site. This analysis involved 97 amino acid sequences, 84 from Eggplant genome [12] and 13 from known anthocyanin related bHLH in other species. The AN1 and the JAF13 clade described in the text are marked with orange and purple triangle, respectively.

A total of 84 gene sequences encoding for bHLHs were retrieved in the eggplant genome and manually inspected, while 159 and 124 were previously identified in tomato [36] and potato [37] genomes respectively. The eggplant bHLHs were then used to construct a phylogenetic tree based on the NJ method (Fig 1) together with 13 known plant proteins related to anthocyanin synthesis belonging to the subgroup IIIf.

Among eggplant proteins, two bHLH factors, i.e. *SMEL*008g319200.1 and the *SMEL*009g326640.1 showed high homology with the major plant bHLH factors belonging to subgroup IIIf. These sequences were previously identified by Barchi et al. [12] and named *SmelJAF13* and *SmelAN*1 in accordance to their homology with tomato Sl*JAF1*3 and Sl*AN1*, respectively. The two proteins fall into distinct evolutionary sub-clades of bHLH involved in anthocyanin regulation of which one comprises members such as JAF13 (tomato, eggplant, petunia and related sequences of other species) and GL3/EGL3 from *A*. *thaliana*, while the other includes members such as AN1 (tomato, eggplant, petunia, and related sequences in other species) and TT8 from *A*. *thaliana* (Fig 1). The Zm*IN1* gene has been considered as a separate group by several authors, due to its unique intron-exon structure [17]. However, no further studies have been performed to elucidate this putative evolutionary divergence. Depending on the species, the two bHLHs are not functionally redundant and they do not complement each other. Indeed, they might regulate anthocyanins synthesis in a specific time and space manner. In tomato and petunia, both JAF13 and AN1 proteins appear to be involved in anthocyanin regulations although in a different manner [17,19].

A similar approach was applied to identify eggplant anthocyanin related MYBs protein encoding genes. The latter were identified by screening the genome of *S*. *melongena* breeding line 67/3 [12] with 25 known plant MYBs (S2 File) related to anthocyanin synthesis belonging to subgroup 4–7 according to the classification by Liu et al [38]. Interestingly, an analogous number of MYB proteins, *i*.*e*. 127, were identified in tomato [39] while 159, were detected in potato [40].

**Fig 2:**
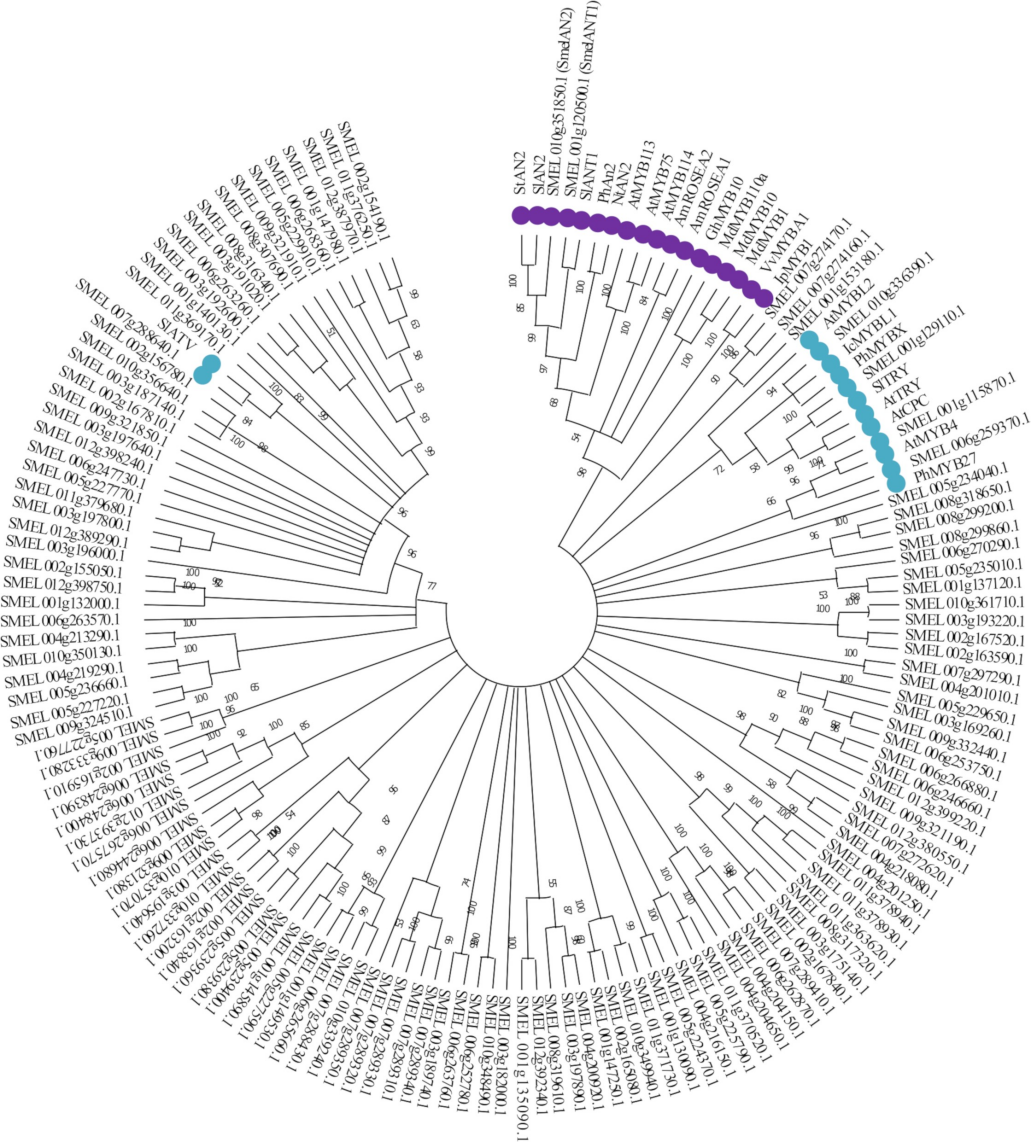
Phylogenetic tree of MYB transcription factors in *S*. *melongena* genome. The optimal NJ tree with the sum of branch length = 45.96037176 is shown. The tree is drawn to scale, with branch lengths in the same units as those of the evolutionary distances used to infer the phylogenetic tree. The percentage of replicate trees in which the associated taxa clustered together in the bootstrap test (1,000 replicates) are shown next to the branches. Branches corresponding to partitions reproduced in less than 50% bootstrap replicates are collapsed. The evolutionary distances were computed using the p-distance method and are in the units of the number of amino acid differences per site. This analysis involved 154 amino acid sequences, 129 from Eggplant and 25 from known anthocyanin related MYBs in other plant species. Clades containing MYB proteins involved in positive and negative anthocyanin regulation are marked with purple and blue circles, respectively.

The identified eggplant MYBs, as well as 25 related MYBs detected in other plant species, were used to construct a phylogenetic tree using the NJ method together (Fig 2). Based on similarity with the R2R3-MYB proteins PhAN2, SlAN2 and SlANT1, which are known to be involved in anthocyanin regulation in petunia and tomato, two homologs were identified in eggplant: *SMEL*010g351850.1, corresponding to *Smel*AN2, and *SMEL*001g120500.1 ortholog to *Smel*ANT1, both of which were previously annotated as putative candidate regulatory MYB in eggplant [12,20]. Moreover, a number of MYB domain encoding proteins were found in evolutionary subgroups closely related to the ones of known anthocyanins regulators. Interestingly, a cluster of 7 R2R3-MYBs, including *SMEL*007g274170.1 and its paralogous genes *SMEL*007g274160.1, *SMEL*001g153180.1, *SMEL*005g234040.1, *SMEL*008g299860.1, *SMEL*008g299200.1 and *SMEL*008g318650.1, might represent other flavonoids regulatory proteins.

**Fig 3:**
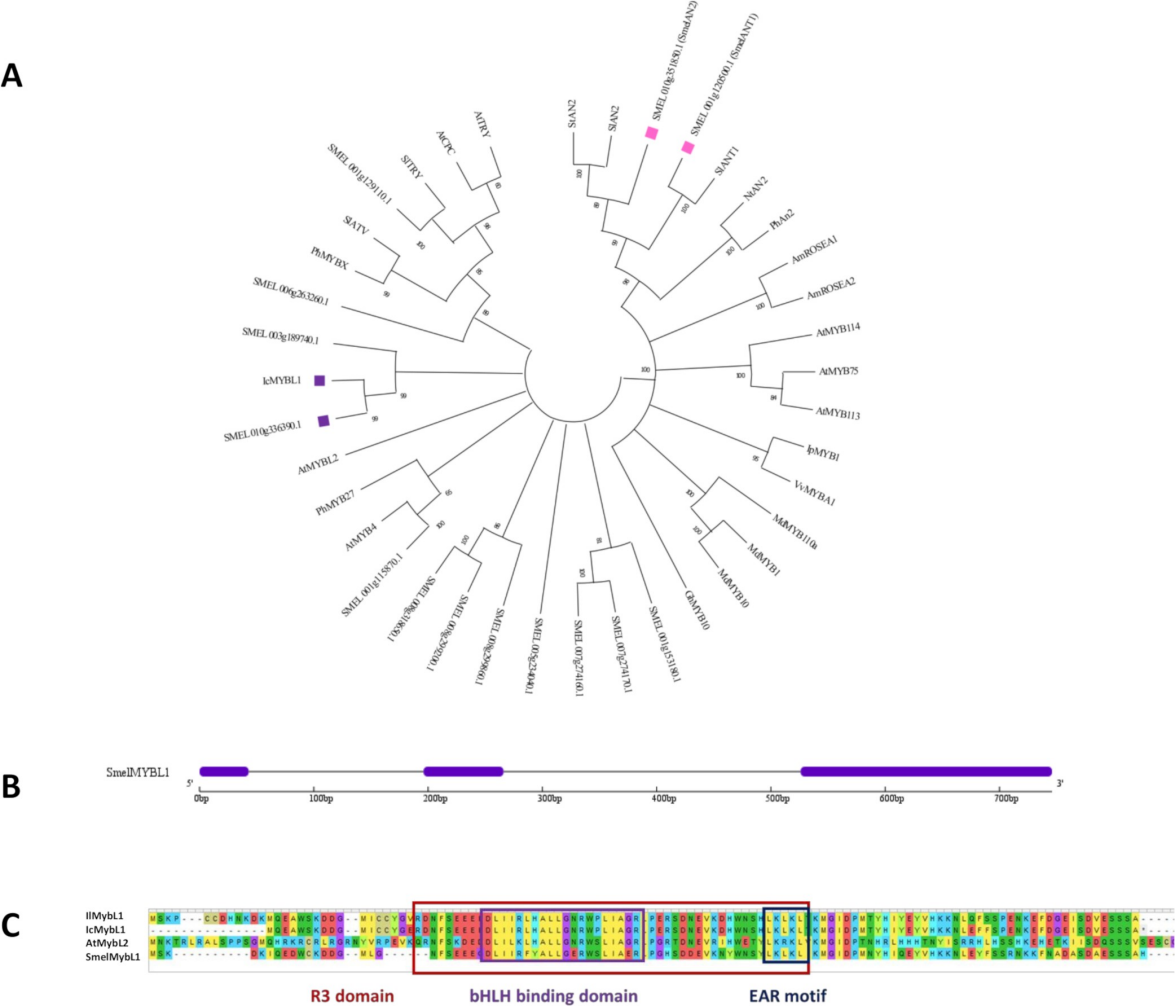
**(A)** Phylogenetic tree of MYB transcription factors related to flavonoid synthesis. The optimal NJ tree with the sum of branch length = 8,61587540 is shown. The tree is drawn to scale, with branch lengths in the same units as those of the evolutionary distances used to infer the phylogenetic tree. The percentage of replicate trees in which the associated taxa clustered together in the bootstrap test (1,000 replicates) are shown next to the branches. Branches corresponding to partitions reproduced in less than 50% bootstrap replicates are collapsed. The evolutionary distances were computed using the p-distance method and are in the units of the number of amino acid differences per site. The analysis included 39 amino acid sequences. Positive and negative candidate MYBs analysed in this work are marked with pink and purple square, respectively. (**B)** Exon/intron structure of *S*. *melongena* MYBL1 gene. The exons and introns are represented by purple boxes and black lines, respectively. (**C)** Domain structure of MYBL1 type repressors.

Several putative repressors of anthocyanin biosynthesis were identified in eggplant. As depicted in Figs 2 and 3A, we identified three clades corresponding to different repressor types: (i) a clade including CPC of three repeat binding domain R3 repressors, which comprises *A*. *thaliana* TRY and CPC, tomato ATV [25,41] and TRY, the putative *S*. *melongena* ortholog of TRY, *Smel*001g129110.1, and petunia MYBX; (ii) a MYBL1 clade of R3 repressors, including *Iochroma* MYBL1 and its eggplant ortholog, *Smel*010g336390.1, *A*. *thaliana* MYBL2, and (iii) a clade including R2R3 MYB repressors belonging to subgroup 4, composed by petunia MYB27, *A*. *thaliana* MYB4 and its eggplant ortholog *Smel*001g115870.1.

According to previous works [42,43], in eggplant two anthocyanin related QTLs are located on chromosome 5 and 10, which respectively explain the type and intensity of anthocyanin pigmentation. We focused our attention on the candidate repressor mapping on Chr10, *SMEL*010G336391.1 (*Smel*MYBL1), whose intron-exon structure is reported in Fig 3B. Similarly to what observed for *Iochroma* MYBL1, *Smel*MYBL1 lost the EAR motif characteristic of the *MYB3like* genes and *petunia MYB27* [5], but acquired a new EAR motif near the end of the R3 domain (Fig 3C). Moreover, the *Smel*MYBL1 is characterized by the bHLH binding motif in the R3 domain and likely binds with the bHLH transcription factors acting as part of an MBW regulatory complex.

### Transcriptional profiling of anthocyanin related genes

**Fig 4:**
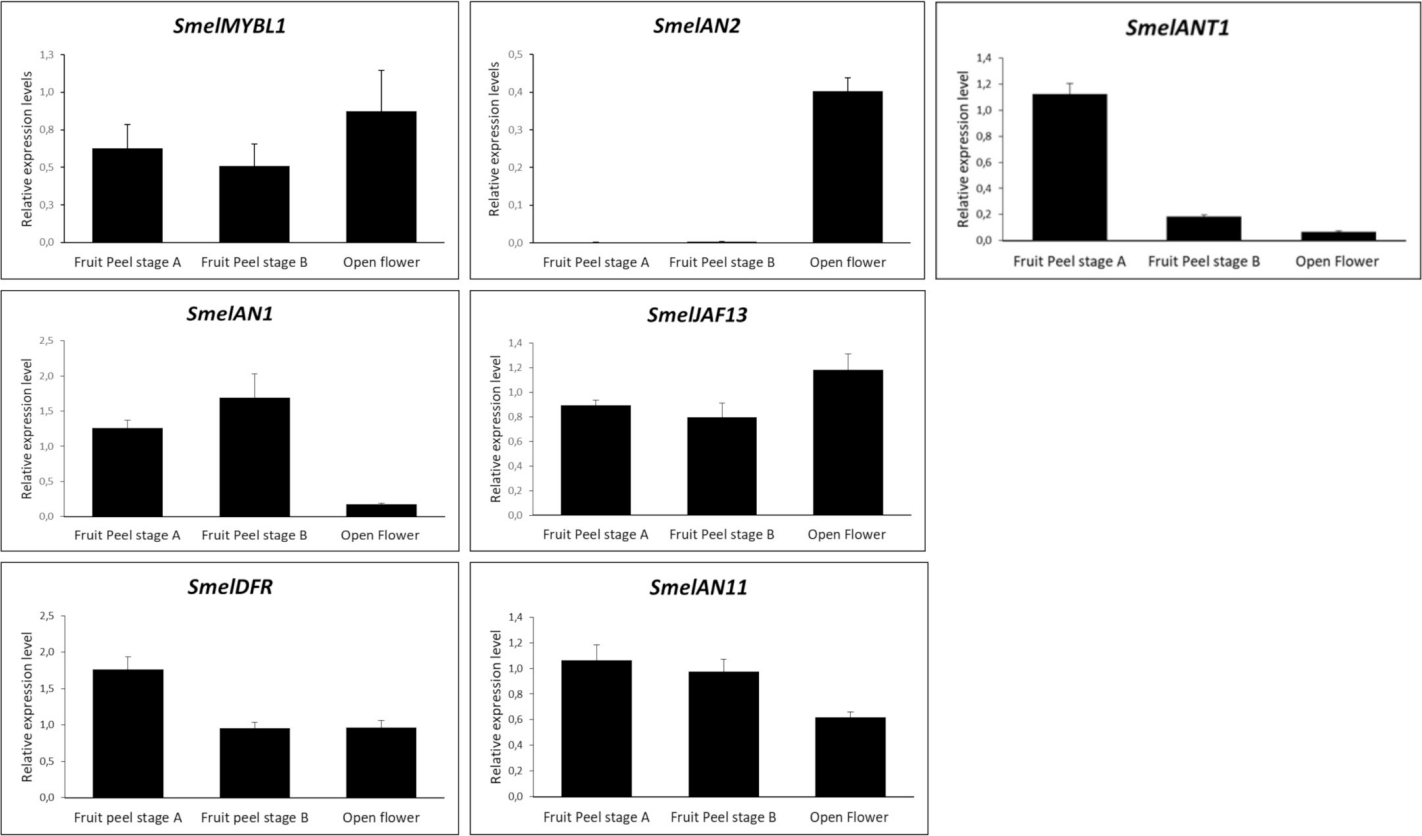
qRT-PCR based transcription profiling of eggplant *MYBL1*, *AN2*, *ANT1*, *AN1*, *JAF13*, *DFR*, *AN11* in two stages of fruit ripening (stage A and B) and in flower organs. Expression levels, measured by qPCR, are shown as relative units using *SmelGAPDH* as reference gene. Data are means of three biological replicates ± SD.

To investigate the function of *SmelANT1*, *SmelAN1*, *SmelAN2*, *SmelJAF13* in the regulation of anthocyanin synthesis, we performed their qRT-PCR expression analysis in flower at the anthesis and fruit at unripe (stage A) and commercial ripening (stage B) (Fig 4). The *SmelAN11* (*SMEL*003G185640.1), a WD40 encoding gene, previously identified by Barchi and colleagues [12], along with *SmelDFR*, a structural gene of the biosynthetic pathway, were also included in the analysis.

As highlighted in Fig 4, *SmelDFR* resulted highly expressed at both stage A and B as well as in flower organs. As previously observed [12], the R2R3 MYB encoding genes *ANT1* and *AN2* showed a tissue specific expression. *AN2* was poorly expressed in all the tissues except flowers, while *ANT1* was highly expressed at the fruit stages A and B (Fig 4). These results reinforce the hypothesis of *AN2* and *ANT1* involvement in the regulation of anthocyanin synthesis in fruits and flowers respectively. Indeed also *SlANT1* and *SlAN2* in tomato [19] as well as *ScAN1* and *ScAN2* in *S*. *commersonii* [44] were found to be differently involved in anthocyanin regulation. The putative repressor encoding gene, *SmelMYBL1*, resulted to be expressed in all analysed tissues.

As previously reported [15,19], the WD40 encoding gene AN11 was found to be constitutively expressed in all the tissues analysed.

The two bHLH encoding genes, *SmelAN1* and *SmelJAF13*, were always expressed in tissues containing anthocyanins, with the former more expressed in fruits and the latter in flower organs (Fig 4). This suggests that multiple MYB-bHLH-WD40 complexes exert their regulatory role in different organs as highlighted in other species [45].

All our qPCR analyses in eggplant tissues and organs confirm a clear correlation of DFR and R2R3 MYB TFs transcript levels with anthocyanin content, as previously reported in eggplant (S3 Fig) [12] as well as other species [9,46,47].

### Yeast two-hybrid

**Fig 5:**
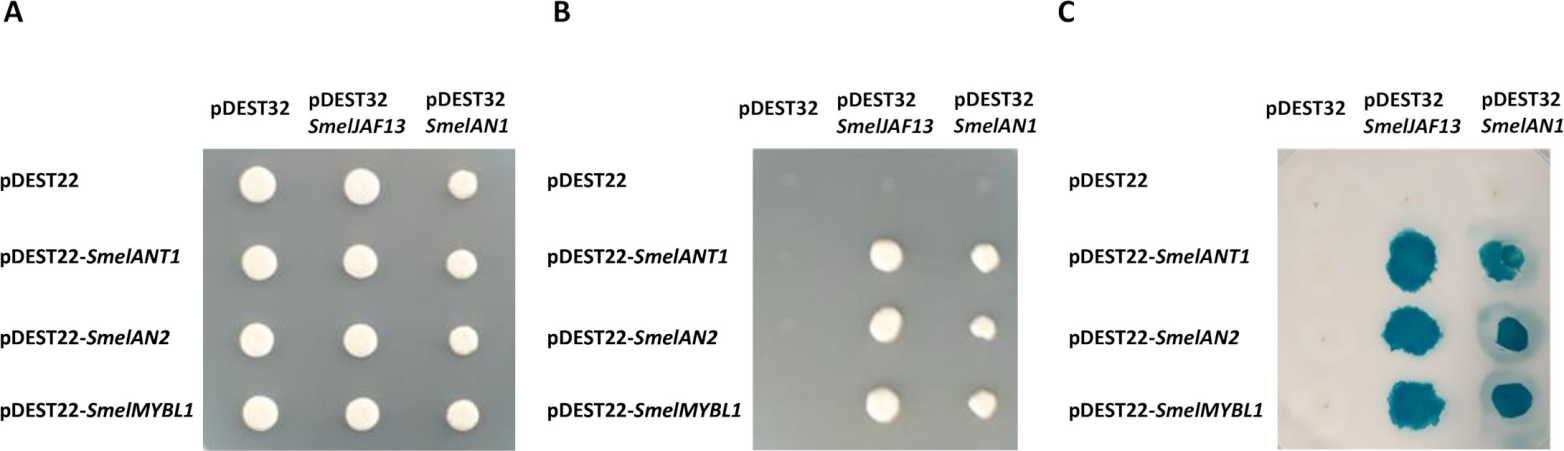
Y2H assay. ANT1, AN2 and MYBL1 were cloned in the prey plasmid pDEST22 and transformed with the bait plasmid pDEST32 (containing JAF13 and AN1). pDEST22 and pDEST32 were used as a negative control. Yeast cells were grown for three days on (A) synthetic complete medium lacking tryphtophan and leucine (-W/-L), (B) on selective medium lacking tryptophan, leucine and uracil (-W/-L/-U) and (C) on Whatman 541 filter papers, saturated 2% X-gal solution.

Interactions between proteins belonging to MYB (*Smel*ANT1, *Smel*AN2 and *Smel*MYBL1) and bHLH (*Smel*JAF13, *Smel*AN1) families were investigated by means of a yeast two-hybrid assay (Fig 5). Since the fusion of anthocyanin MYB regulators with the GAL4 binding domain led to the auto activation of reported genes, these were associated to GAL4 activation domain. Thus, the coding sequence of *Smel*JAF13 and *Smel*AN1 were inserted in the bait vector, while *Smel*ANT1, *Smel*AN2 and *Smel*MYBL1 in the prey vector.

Yeast cells co-transformed with *Smel*ANT1, *Smel*AN2 and *Smel*MYBL1 in combination with *Smel*JAF13 or *Smel*AN1 grew on selective medium lacking leucine, tryptophan and uracil (Fig 5B), demonstrating the ability of different MYB proteins to form a complex with bHLH partners. Negative controls, consisting of yeast cells co-transformed with prey plasmids containing MYB proteins and empty bait plasmid, as well as the opposite combination (*i*.*e*. bait plasmids containing bHLH proteins and empty pray plasmid), did not grow on selective medium, indicating the lack of interaction. Our findings were further supported by the formation of blue colonies through β-galactosidase assay (Fig 5C). These results are in accordance with those reported by D’Amelia et al. [48], which highlighted the ability of StAN1 (ortholog of *Smel*ANT1) to interact with StbHLH1 and StJAF13. Besides, the protein interaction assay carried out in tomato protoplasts demonstrated that SlMYB-ATV could actually bind both the endogenous bHLH factors SlAN1 and SlJAF13 [25], in analogy with *Smel*MYBL1.

### Bimolecular Fluorescent Complementation (BiFC)

**Fig 6:**
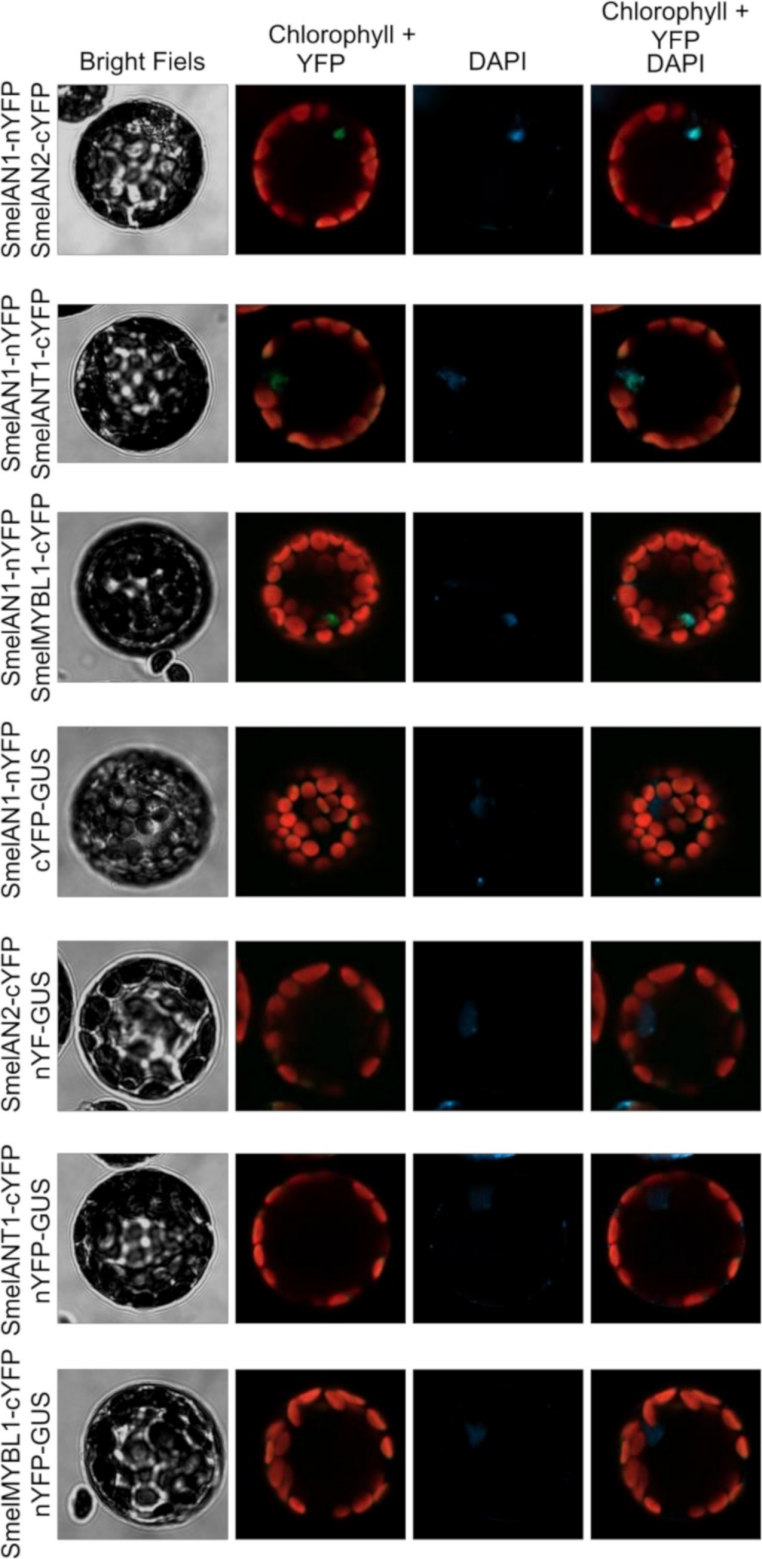
Bimolecular fluorescent complementation assay. *Smel*AN1-nYFP fusion proteins was co-expressed transiently with *Smel*AN2-cYFP, *Smel*MYBL1-cYFP or *Smel*ANT1-cYFP fusion proteins in freshly isolated Arabidopsis mesophyll protoplasts. GUS protein fused to both nYFP or cYFP was used as negative control. The cellular localization of interactions was investigated through DAPI staining.

To confirm the interaction between AN1 and MYBs proteins, we carried out a Bimolecular Fluorescent Complementation experiment using *A*. *thaliana* mesophyll protoplasts as model system. The N-terminal domain of the Yellow fluorescent protein (nYFP) was fused in frame with the *Smel*AN1 CDS missing of the stop codon, while the CDSs of the three investigated MYBs, after removal of the stop codons, where fused upstream the C-terminal domain of the YFP (cYFP). Freshly isolated *Arabidopsis* protoplasts were then transformed with combinations of plasmids carrying the expression cassette for the fusion proteins and subjected to confocal microscopy the following day. A fluorescent signal, indicating an interaction between the investigated proteins, was reported for all the combinations tested (Fig 6), thus confirming the ability of *Smel*AN1 to interact with *Smel*AN2, *Smel*ANT1 and *Smel*MYBL1. Otherwise, no signal was detected in the control transformations (Fig 6). By means of DAPI staining, we also proved that all complexes localized into the nuclei.

### Transient heterologous expression in *Nicotiana benthamiana*

**Fig 7:**
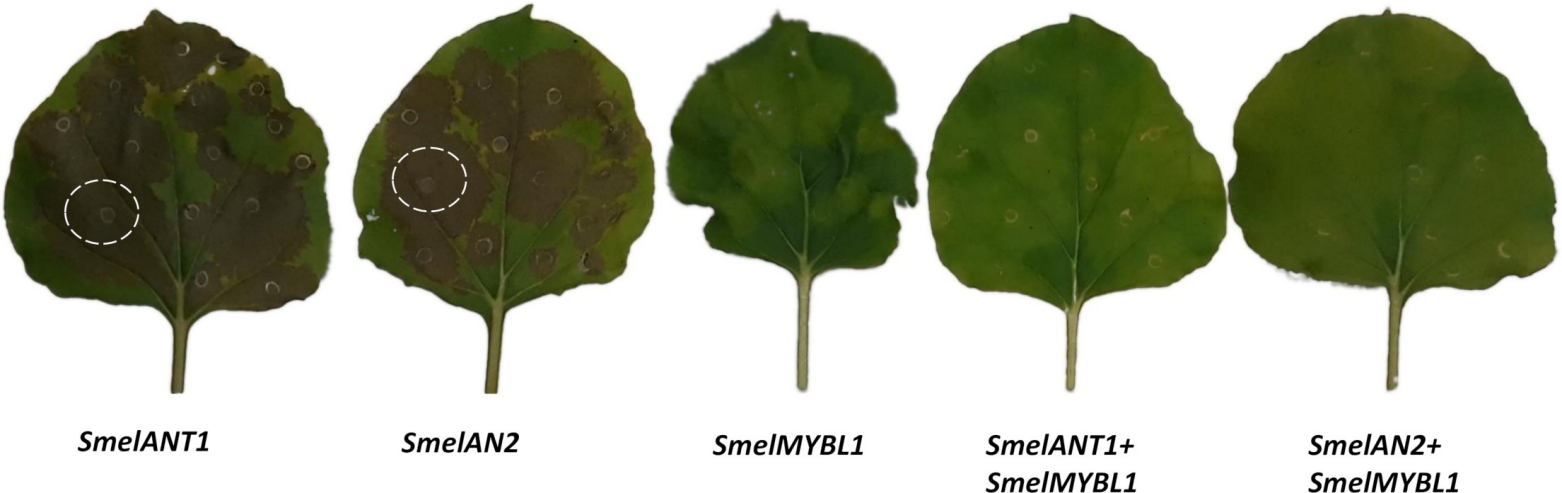
The effects of over-expression of ANT1, AN2 and MYBL1 in *Nicotiana benthamiana*. Leaves of *N*. *benthamiana* after agroinfiltration with ANT1, AN2 and MYBL1 and a combination of MYBL1 with ANT1 or AN2. Anthocyanin accumulation is indicated by dotted white circles.

To verify the effect of ectopic expression of the three *Smel*MYBs and two *Smel*bHLHs *in planta*, we carried out a *N*. *benthamiana* leaf transient expression assay (Fig 7). *Agrobacteria* transformed with a pEAQ expression vector containing *SmelANT1*, *SmelAN2*, *SmelMYBL1*, *SmelJAF13*, *SmelAN1* were infiltrated individually or in combination (*MYBL1* with *ANT1* or *AN2*) in *N*. *benthamiana* leaves. Plants agro-infiltrated with the empty vector were used as negative controls. Four days after infiltration, an anthocyanin-pigmented phenotype was clearly visibly in pEAQ_ANT1 and pEAQ_AN2 agro-infiltrated leaves, while no anthocyanin accumulation was detected upon expression of MYBL1 as well as of JAF13 and AN1 (S4 Fig). Interestingly co-expression of MYBL1 together with ANT1 and AN2 prevented anthocyanin accumulation.

**Fig 8:**
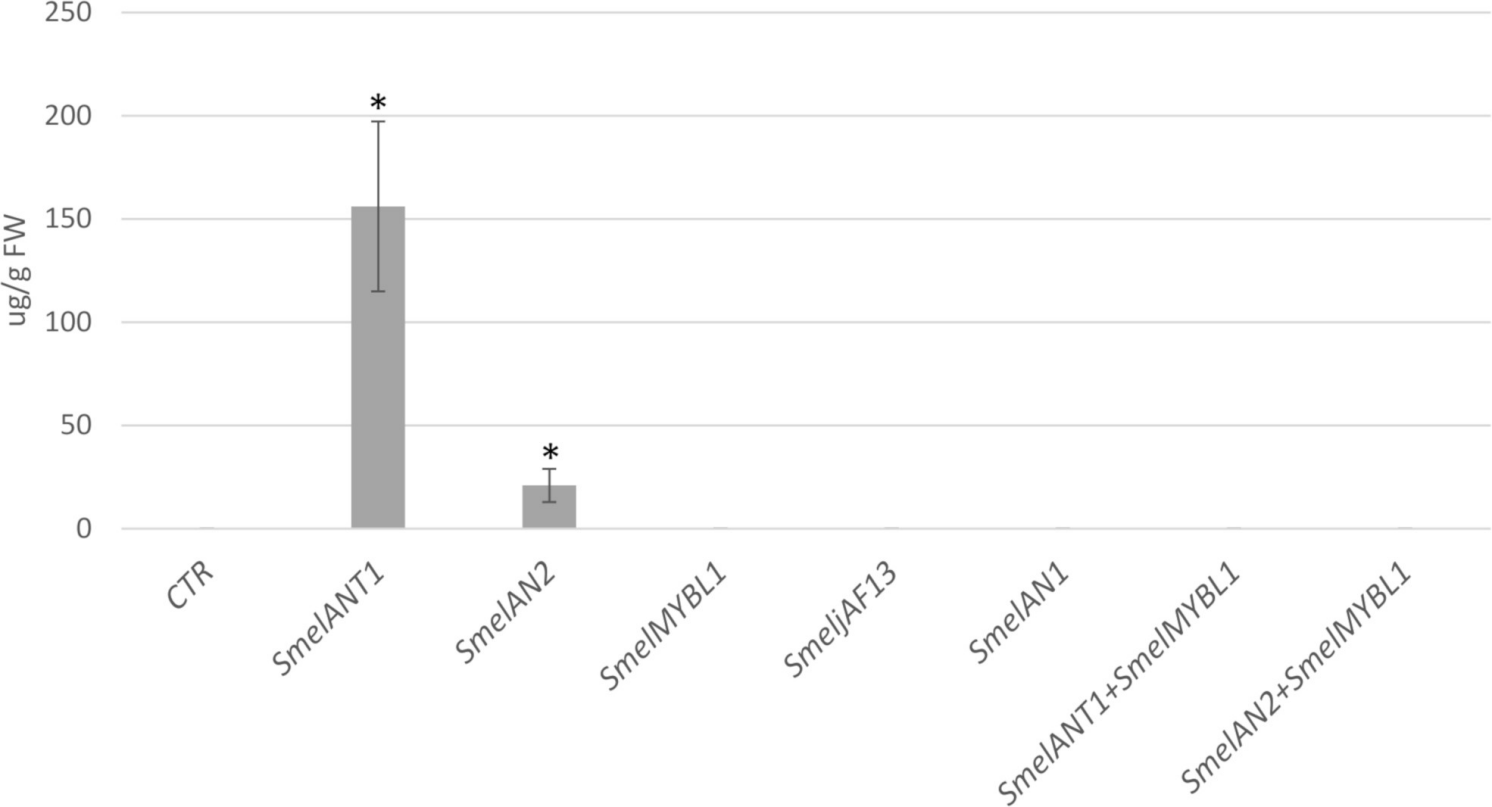
The effects of over-expression of ANT1, AN2 and MYBL1 in *Nicotiana benthamiana*. Concentration of delphinidin 3-*O*-rutinoside in tissue extracts of *N*. *benthamiana* control (CTR) and transiently transformed leaves (ANT1, AN2, AN1, JAF13, MYBL1 and a combination of MYBL1 with ANT1 or AN2). Error bars represent SD (n = 3). Asterisk indicates significance based on Tukey’s test (P≤0.05).

HPLC analyses on the transformed leaves were also carried out to provide additional evidence of anthocyanin accumulation (Fig 8). Indeed *N*. *benthamiana* leaves transiently expressing *Smel*ANT1 and *Smel*AN2 were found to accumulate 156.14 and 20.92 μg/g fresh weight (FW) respectively of delphinidin 3-*O*-rutinosid, which was not detectable in leaves agroinfiltared with the empty vector (Fig 8). In line with the visual observation, no delphinidin 3-*O*-rutinosid was detected in samples agroinfiltrated with MYBL1, JAF13 and AN1 as well after co-infiltration of MYBL1 together with *Smel*ANT1 and *Smel*AN2. Our results thus support previously transient expression in tobacco [20] and stable expression in eggplant [9,21], in which anthocyanin accumulation was verified for *Smel*ANT1. Co-expression of MYBL1 together with ANT1 prevented completely the anthocyanin accumulation, acting as a negative regulator of the biosynthetic pathway. Analogous results were found in *N*. *tabacum* after the expression of *I*. *loxense* MYBL1, resulting in a nearly complete loss of floral anthocyanins in [27]. On the other side, the co-expression of ANT1 together with *Smel*MYB44/*Smel*MYB86, proposed as a negative regulators of the anthocyanin pathway, led to a decrease but not to a complete stoppage of anthocyanin production in eggplant [10].

## Conclusion

A model has been designed to describe the regulation network of anthocyanin biosynthesis in plant [5]. Feedback inhibition of anthocyanin biosynthesis is caused by the interaction of R3 MYB repressors with the core MBW activation complex [25, 41, 49] The replacement of one of the R2R3 MYB partners in the MBW complex with an R3 MYB may transform the complex from an activator to a repressor of anthocyanin gene transcription.

We identified in eggplant the candidate TFs in the anthocyanin related MBW activation complexes. These include two MYB TFs (*Smel*ANT1 and *Smel*AN2), two bHLH TFs (*Smel*JAF13 and *Smel*AN1) and one WDR (*Smel*AN11). *Smel*MYBL1, which belongs to R3 MYB, might represent a new component of the eggplant MBW complex. The latter appears to act as inhibitor of MBW complex by competing with MYB activators (*Smel*ANT1 and *Smel*AN2) for binding to *Smel*JAF13 and *Smel*AN1, thus hindering the chances to form new MBW complexes.

In recent years the CRISPR/Cas9 system has emerged as a powerful technology for genome editing and is now widely used to explore gene function. Thanks to the ongoing development of this technology in eggplant [50], our future goal will be to deepen the functional characterization of the isolated genes and validate MBW activation complex in the species. The anthocyanin accumulation in eggplant berries is determined by the balance between biosynthesis and degradation, thus our increase in understanding the genetic mechanisms regulating both processes may open the way for future genetic engineering approaches aimed increase the content of fruit anthocyanins through increasing their production but also through reducing their degradation.

## Supporting information

S1 File Evolutionary relationships of bHLH proteins. (TXT)

S2 File Evolutionary relationships of MYB proteins. (TXT)

S1 Fig Stages of the eggplant flower and fruit employed: A) Open flowers; B) Fruits Ø 2–4 cm at 8–14 DAF (named stage A); C) Fruits at commercial ripening (named stage B) at approximately 38 DAF. Scale bar in each image represents 1cm.
(TIFF)

S2 Fig Calibration curve of delphinidin 3-*O*-rutinoside standard (50, 20, 10, 5, 2, 1 μg/mL). (TIFF)

S3 Fig Expression value (FPKM) in 5 eggplant tissue samples: root, leaf, flower, fruits stage A (unripe), fruit stage B (commercial ripening) redrawn from data from Barchi et al., 2019 [12]. The average value is sorted by colour, from yellow (low) to blue (high).
(TIFF)

S4 Fig The effects of over-expression of JAF13 and AN1 in *Nicotiana benthamiana*. Leaves of *N*. *benthamiana* after agroinfiltration with JAF13 and AN1.
(TIFF)

S1 Table List of primers used in this study. (DOCX)
