# Correction: Identification of a new R3 MYB type repressor and functional characterization of the members of the MBW transcriptional complex involved in anthocyanin biosynthesis in eggplant (*S*. *melongena* L.)

**DOI:** 10.1371/journal.pone.0235081

**Published:** 2020-06-17

**Authors:** The PLOS ONE Staff

The authors’ first and last names are incorrectly switched throughout the article. The publisher apologizes for the error. Please view the correct author byline, citation, and author contributions here:

Andrea Moglia, Francesco Elia Florio, Sergio Iacopino, Alessandra Guerrieri, Anna Maria Milani, Cinzia Comino, Lorenzo Barchi, Arianna Marengo, Cecilia Cagliero, Patrizia Rubiolo, Laura Toppino, Giuseppe Leonardo Rotino, Sergio Lanteri, Laura Bassolino

Moglia A, Florio FE, Iacopino S, Guerrieri A, Milani AM, Comino C, et al. (2020) Identification of a new R3 MYB type repressor and functional characterization of the members of the MBW transcriptional complex involved in anthocyanin biosynthesis in eggplant (*S*. *melongena* L.). PLoS ONE 15(5): e0232986. https://doi.org/10.1371/journal.pone.0232986

**Conceptualization:** Andrea Moglia, Cinzia Comino, Laura Bassolino.

**Data curation:** Andrea Moglia, Sergio Iacopino, Lorenzo Barchi, Arianna Marengo, Cecilia Cagliero, Patrizia Rubiolo, Laura Bassolino.

**Investigation:** Andrea Moglia, Francesco Elia Florio, Sergio Iacopino, Alessandra Guerrieri,

Anna Maria Milani, Lorenzo Barchi, Arianna Marengo, Laura Toppino, Laura Bassolino.

**Supervision:** Andrea Moglia, Laura Bassolino.

**Writing–original draft:** Andrea Moglia, Laura Bassolino.

**Writing–review & editing:** Andrea Moglia, Francesco Elia Florio, Cinzia Comino, Lorenzo Barchi, Laura Toppino, Giuseppe Leonardo Rotino, Sergio Lanteri, Laura Bassolino.
