# Supplementary figures and images for: Identification of a new R3 MYB type repressor and functional characterization of the members of the MBW transcriptional complex involved in anthocyanin biosynthesis in eggplant (S. melongena L.)
Source: PLoS One. 2020 May 14;15(5):e0232986. doi: 10.1371/journal.pone.0232986 (PMC7224497; doi:10.1371/journal.pone.0232986)

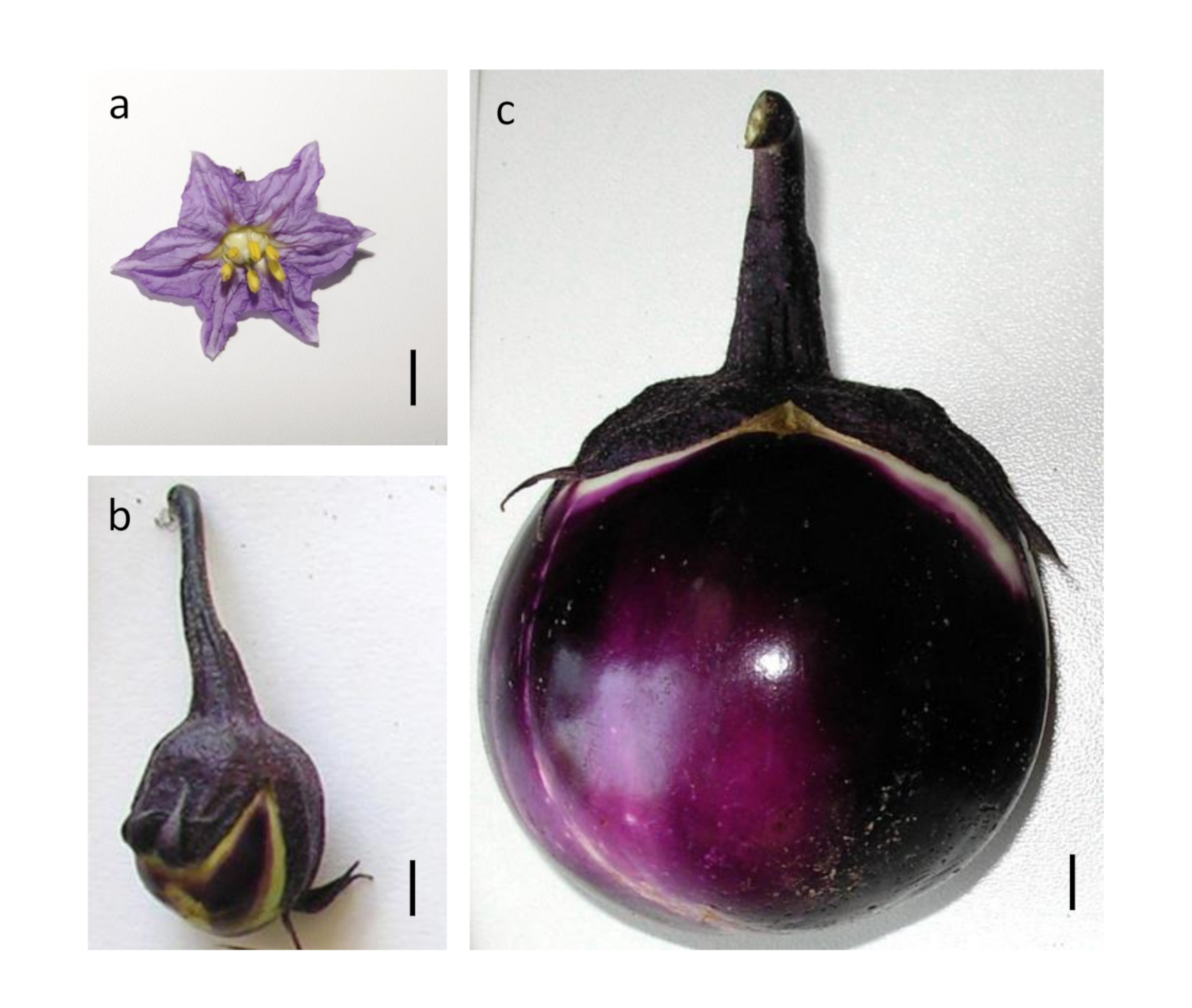

Supplement: S1 Fig — Stages of the eggplant flower and fruit employed: A) Open flowers; B) Fruits Ø 2–4 cm at 8–14 DAF (named stage A); C) Fruits at commercial ripening (named stage B) at approximately 38 DAF. Scale bar in each image represents 1cm. (TIFF) [file pone.0232986.s003.tiff]

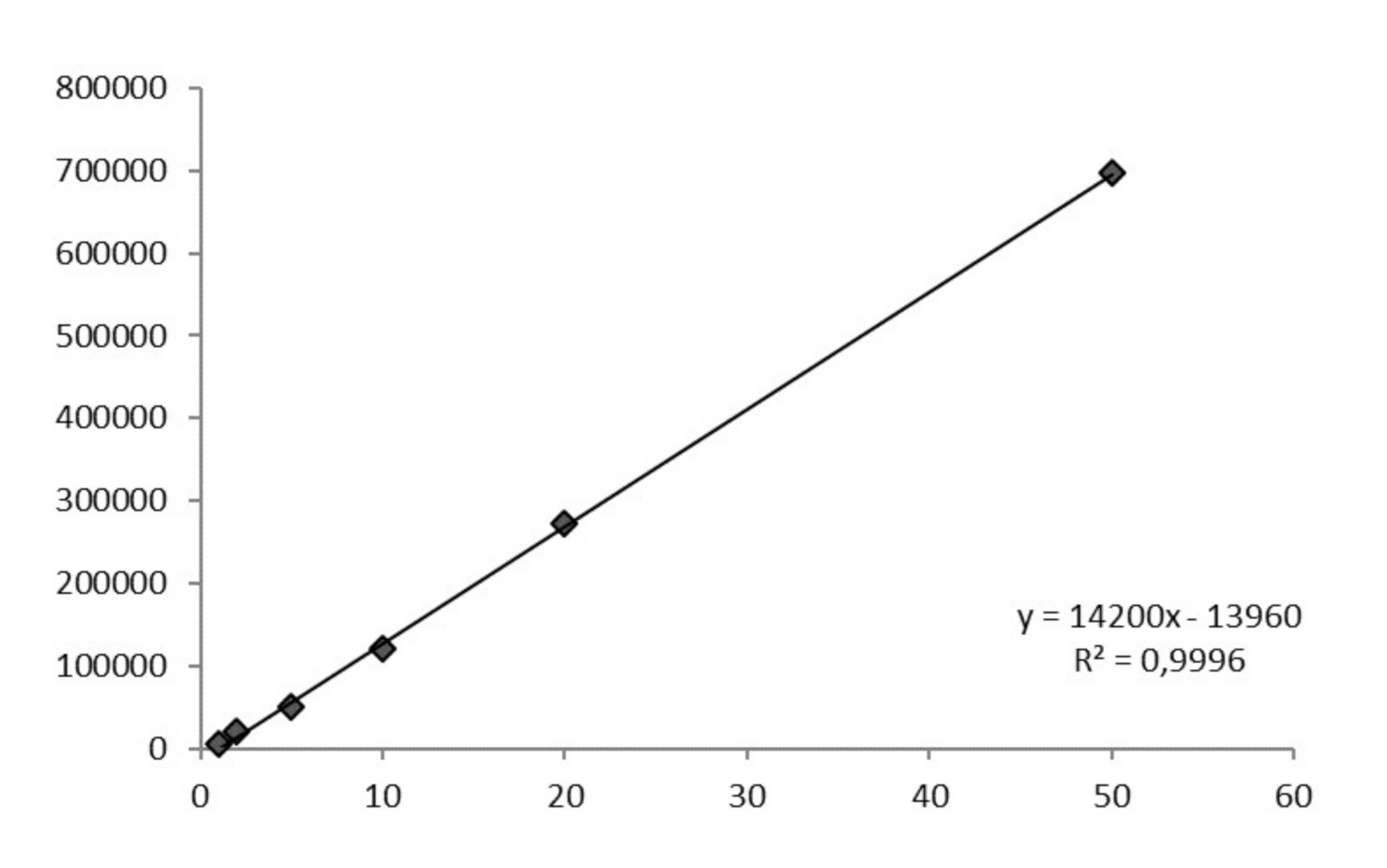

Supplement: S2 Fig — (TIFF) [file pone.0232986.s004.tiff]

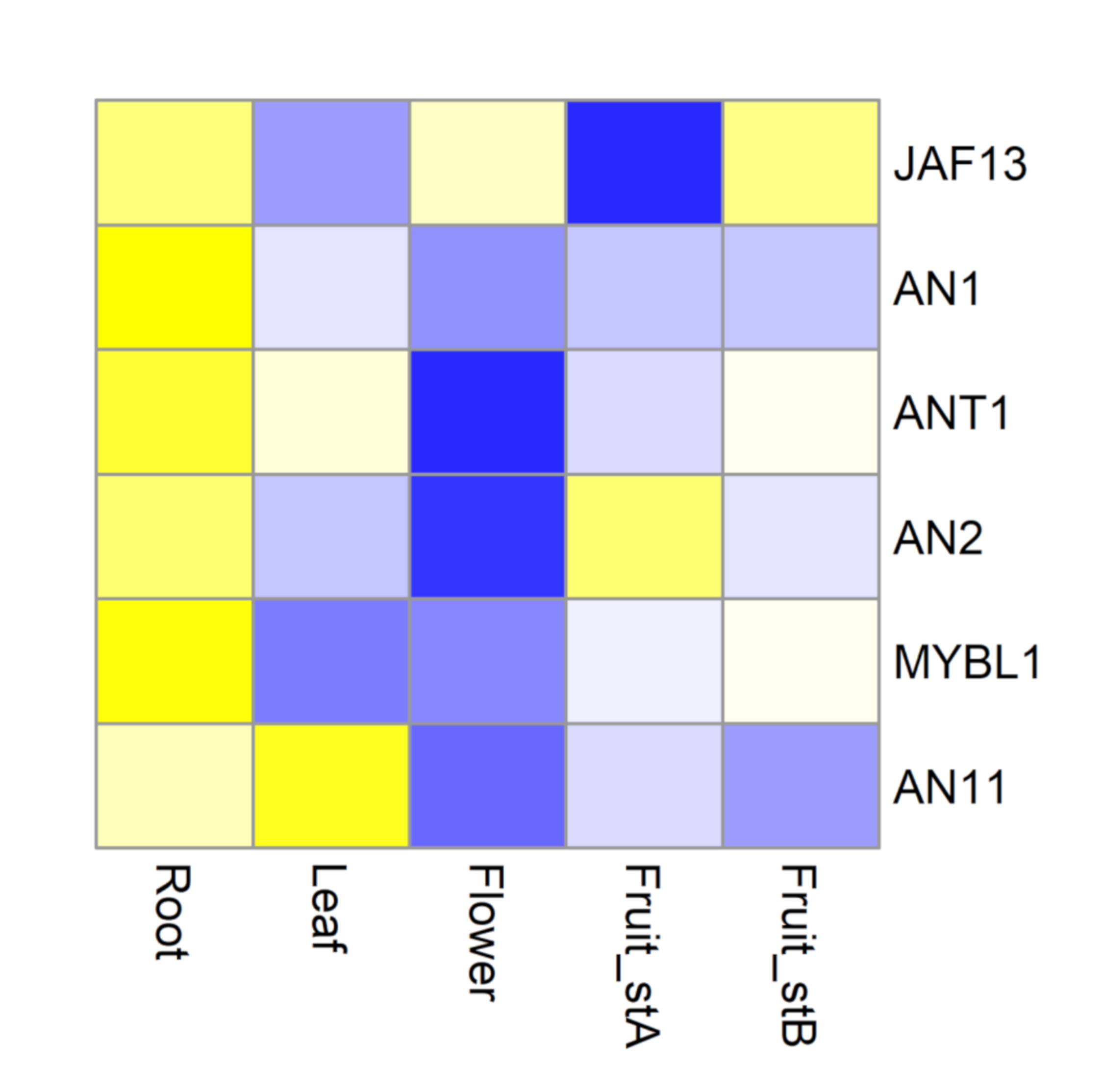

Supplement: S3 Fig — Expression value (FPKM) in 5 eggplant tissue samples: root, leaf, flower, fruits stage A (unripe), fruit stage B (commercial ripening) redrawn from data from Barchi et al., 2019 [12]. The average value is sorted by colour, from yellow (low) to blue (high). (TIFF) [file pone.0232986.s005.tiff]

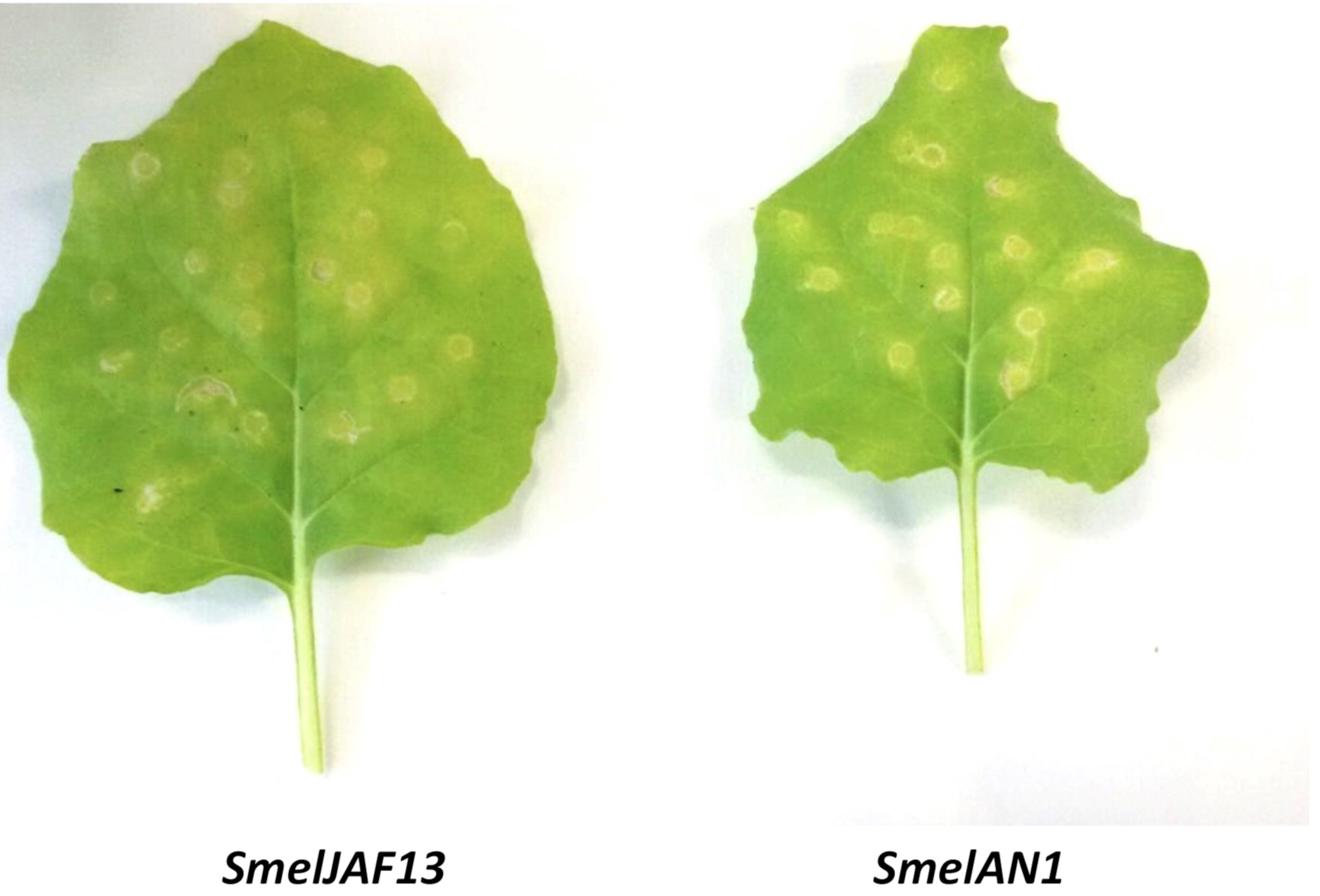

Supplement: S4 Fig — Leaves of N. benthamiana after agroinfiltration with JAF13 and AN1. (TIFF) [file pone.0232986.s006.tiff]
