# Supplementary material for: Identification of a new R3 MYB type repressor and functional characterization of the members of the MBW transcriptional complex involved in anthocyanin biosynthesis in eggplant (S. melongena L.)
Source: PLoS One. 2020 May 14;15(5):e0232986. doi: 10.1371/journal.pone.0232986 (PMC7224497; doi:10.1371/journal.pone.0232986)
Supplement: S1 Table — (DOCX) [file pone.0232986.s007.docx]

**S1 Table: List of primers used in this study.**

| **Gene** | **Sequences** | **Scope** |
| --- | --- | --- |
| ***SmelANT1*** | FW 5’- ggaccgcaaacgatgtaaag-3’  RV 5’- tttccgaggttgaggtcttatt-3’ | qRT-PCR |
| ***SmelAN2*** | FW 5’-aacgccacaaagaaattgga-3’  RV 5’-ccagcaaacttgtccaccat-3’ | qRT-PCR |
| ***SmelAN1*** | FW 5’- ttgcccagacggtagaac-3’  RV 5’- cagccgacccaaccccactt-3’ | qRT-PCR |
| ***SmelJAF13*** | FW 5’-actgttcaggcaggggaagt-3’  RV 5’-cctgtggttgtatgtcgtct-3’ | qRT-PCR |
| ***SmelAN11*** | FW 5’-actgtgcccctttgacctct-3’  RV 5’-attgggtttcgacagcccc-3’ | qRT-PCR |
| ***SmelMYBL1*** | FW 5’-ccttggtgaaagatggtcgc-3’  RV 5’-ggaactttcagcgtcagaagc -3’ | qRT-PCR |
| ***SmelDFR*** | FW 5’-atacaaacttaacgctgtggaaag-3’  RV 5’-agtcgctccagctagtctcgtca-3’ | qRT-PCR |
| ***SmelGAPDH*** | FW 5’- ggtgccaagaaggttgtgat-3’  RV 5’- ccaatgctagttgcacaacg-3’ | qRT-PCR |
| ***SmelAN2*** | FW 5’-caccatgaatactgctactgttgctaagtca-3’  RV 5’-cccctaattaaatagattccataggtca-3’ | Gateway cloning |
| ***SmelMYBL1*** | FW 5’-caccatgagcaaggataaaatacaag-3’  RV 5’-ttaatgtgcagaggaactttcag-3’ | Gateway cloning |
| ***SmelANT1*** | FW 5’ggggacaagtttgtacaaaaaagcaggctcgatgaataatcctcctataatctgtacgtctg-3’  RV 5’ ggggaccactttgtacaagaaagctgggtccttaatcaagtaaattccataaatcaatatca-3’ | Gateway cloning |
| ***SmelJAF13*** | FV 5’- ggggacaagtttgtacaaaaaagcaggctcgatggctatgggacaccaag-3’  RV 5’- ggggaccactttgtacaagaaagctgggtcctcaagatttccaaactgctctc-3’ | Gateway cloning |
| ***SmelAN1*** | FV 5’- ggggacaagtttgtacaaaaaagcaggctcgatgatggagatcatacagcctag-3’  RV 5’- ggggaccactttgtacaagaaagctgggtccttaaactctagggattatctgatgt-3’ | Gateway cloning |
